# Levels of Anxiety and Depression Before Palliative Reirradiation Are Comparable to Those Before First Palliative Radiotherapy

**DOI:** 10.7759/cureus.2519

**Published:** 2018-04-23

**Authors:** Carsten Nieder, Thomas A Kämpe

**Affiliations:** 1 Dept. of Oncology and Palliative Medicine, Nordland Hospital Trust

**Keywords:** palliative radiotherapy, re-irradiation, cancer, anxiety, depression, edmonton symptom assessment system

## Abstract

Introduction: The purpose of this study was to evaluate whether or not patients scheduled for the reirradiation of a previously treated target volume report reduced levels of anxiety and depression, compared to patients receiving their first course of palliative radiotherapy, e.g., because they are familiar with the process of treatment planning and delivery.

Methods: A retrospective comparison of two groups of patients (37% reirradiated, overall 102 patients), which scored their symptoms before palliative radiotherapy with the Edmonton symptom assessment system (ESAS).

Results: The two groups differed significantly with regard to the incidence of bone metastases, which was higher in the reirradiation group. Mean anxiety and depression scores were not significantly different between the two groups. The same was true for the proportion of patients with symptom scores ≥4. Analyses limited to patients treated for bone metastases revealed no significant differences either. Survival was similar, too.

Conclusion: The facts that similar ESAS scores of anxiety and depression were observed and that prognosis was comparable suggest that the magnitude of these symptoms might be associated with the presence of incurable cancer itself (or the related somatic symptom burden) rather than the setting in which palliative radiotherapy is performed.

## Introduction

For many common indications, palliative radiotherapy can be repeated to a previously irradiated target volume [1-4]. Such reirradiation often results in a clinical benefit, e.g., pain relief. In general, patients scheduled for radiotherapy have reported heightened levels of anxiety and/or depression [5-6], and current studies are looking at different interventions that might reduce these symptoms [7-8]. Relatively few studies have examined quality of life and patient-reported symptoms, including but not limited to anxiety and depression, before palliative reirradiation [2,9-10]. We hypothesized that patients scheduled for the reirradiation of a previously treated target volume might report reduced levels of anxiety and depression, as compared to patients receiving their first course of palliative radiotherapy, because they are familiar with the process of treatment planning and delivery, as well as the facility and staff they are going to meet. In order to test this hypothesis, a retrospective, single-institution study was performed, which employed previously collected questionnaires (Edmonton symptom assessment system, ESAS). This short, one-sheet questionnaire has been used by different radiotherapy departments and addresses major symptoms and wellbeing on a numeric scale of 0-10 (highest symptom severity 10), including anxiety and depression [6,11-13].

## Materials and methods

The study included 102 unselected, consecutive patients at an academic teaching hospital, all of whom received palliative radiotherapy in routine clinical practice during the time period 2013-2015. Since 2013, the standard pre-treatment workup included symptom assessment with the ESAS tool, administered by a registered oncology nurse immediately before oncologist consultation and imaging for treatment planning, i.e., approximately one week before palliative radiotherapy. All medical records, except for inpatient psychiatric care, were available in the hospital’s electronic patient record system. A statistical analysis was performed with IBM SPSS Statistics 24 (IBM, Armonk, NY, US). We analyzed two different subgroups, patients who were reirradiated to a previously treated target volume and those who received their first course of palliative radiotherapy. We employed the chi-square test (when appropriate, the Fisher exact probability test or t-test). A p-value ≤0.05 was considered statistically significant. Two-tailed tests were performed. Actuarial survival from start of radiotherapy was analyzed with the Kaplan-Meier method and log-rank test. Ethical approval was not required for this study in accordance with the national and institutional guidelines.

## Results

Thirty-eight of 102 patients (37%) received palliative reirradiation, mainly for bone metastases. Time from initial cancer diagnosis was significantly longer in the reirradiation group, while most other baseline characteristics, including age and performance status, were comparable to those of patients who received their first palliative radiotherapy (Table 1).

**Table 1:** Baseline characteristics before palliative radiotherapy RT: radiotherapy, ECOG PS: Eastern Cooperative Oncology Group performance status * collapsed into four categories (prostate/breast/lung/other) ** collapsed into four categories (bone/brain/lung/other)

Characteristic	No Reirrad.	%	No 1. RT	%	p-value
Gender					
Male	46	72	29	76	
Female	18	28	9	24	0.65
Primary tumor site					
Prostate	16	25	15	39	
Breast	6	9	6	16	
Lung	20	31	8	21	
Colorectal	3	5	2	5	
Bladder	5	8	0	0	
Malignant melanoma	4	6	0	0	
Others	10	16	7	18	0.23*
RT target type					
Bone metastases	34	53	32	84	
Brain metastases	9	14	2	5	
Lymph node metastases	2	3	0	0	
Lung	10	16	2	5	
Prostate or bladder	7	11	1	3	
Others	2	3	1	3	0.02**
RT fractionation					
>10 fractions	12	19	9	24	
10 fractions	31	48	10	26	
5-9 fractions	17	27	13	34	
<5 fractions	4	6	6	16	0.15
Systemic cancer treatment					
No	17	27	2	5	
Within 4 wk. before RT	30	47	23	61	
More than 4 wk. before RT	17	27	13	34	0.03
Mean time from first cancer diagnosis to RT	42 months		72 months		0.009
Mean ECOG PS	1.8		1.7		0.73
Mean age	71 years		72 years		0.77

Mean anxiety and depression scores were not significantly different between the two groups, as illustrated in Table 2.

**Table 2:** Association between Edmonton symptom assessment system (ESAS) scores and radiotherapy setting RT: radiotherapy

	Reirradiation Mean, SD, range	1. RT Mean, SD, range	
Parameter			p-value
Anxiety	2.5 ± 2.7, 0-8	2.7 ± 3.2, 0-10	0.71
Depression	2.2 ± 2.4, 0-8	2.1 ± 3.0, 0-10	0.86
Pain (not moving)	3.7 ± 2.6, 0-8	2.3 ± 2.5, 0-9	0.01
Pain (moving)	5.3 ± 2.9, 0-9	3.9 ± 3.2, 0-10	0.03
Fatigue	4.7 ± 2.8, 0-9	4.2 ± 3.0, 0-10	0.41
Nausea	0.8 ± 1.3, 0-5	1.4 ± 2.2, 0-8	0.18
Appetite	3.4 ± 3.1, 0-10	4.0 ± 3.4, 0-10	0.34
Constipation	2.9 ± 3.3, 0-10	2.3 ± 3.0, 0-10	0.36
Dry mouth	2.5 ± 2.4, 0-8	3.2 ± 3.0, 0-10	0.21
Dyspnea	2.1 ± 2.5, 0-10	3.0 ± 2.9, 0-10	0.11
Sleep	2.6 ± 2.7, 0-8	2.6 ± 2.8, 0-10	0.90
Overall wellbeing	3.4 ± 2.3, 0-9	3.7 ± 2.7, 0-10	0.56

The same was true for the proportion of patients with symptom scores ≥4. Regarding anxiety, 38% of reirradiated and 31% of first-time-irradiated patients reported scores ≥4 (p = 0.52). Regarding depression, 29% of reirradiated and 25% of first-time-irradiated patients reported scores ≥4 (p = 0.82). Significant differences were seen for two ESAS items only, namely, the two pain-related items (Table 2). 

An analysis limited to patients with bone metastases was also performed because of the significantly different numbers of patients with this treatment indication and the resulting higher pain scores in the reirradiated group. This analysis revealed insignificant differences in mean pain scores, anxiety, and depression between the reirradiation and the first irradiation groups (pain when not moving 3.9 vs. 2.9, pain when moving 5.5 vs. 5.1, anxiety 2.7 vs. 2.7, depression 2.5 vs. 2.8; all p>0.15, respectively).

The actuarial median survival was 6.8 months for reirradiated and 5.5 months for first-time-irradiated patients, p=0.16. Identical proportions of short-term survivors (radiotherapy in the last 30 days of life) were recorded (11% each).

## Discussion

In the present study, the question “do patients who are scheduled to receive palliative radiotherapy report less anxiety and depression if they are to receive reirradiation" was addressed. We hypothesized that previous experience and facing a known environment might be an advantage. However, the data failed to show any significant differences in anxiety and depression between reirradiated and first-time-irradiated patients. For the hypothesis to be true, several assumptions have to be met. For example, the previous experience with palliative radiotherapy must have been positive and the patient must be able to remember this experience. Our retrospective study was not able to shed light on these aspects because this information was not collected when the patients presented for treatment planning and scored their symptoms on the ESAS sheet. If the levels of anxiety and depression reflect worries about the prognosis, especially in patients with very limited survival expectation, radiation-related issues might be of lesser concern. Therefore, we analyzed short-term and median survival. No significant or clinically relevant differences between the two groups were found.

The data revealed imbalances regarding the proportion of patients with bone metastases and, probably as a consequence, pain scores before radiotherapy. More reirradiated patients had bone metastases and their pain scores were higher. In theory, pain might also interfere with anxiety and depression, even if previous studies showed no strong correlations [11,13-14]. To correct for this potential source of bias, we excluded patients treated for indications other than bone metastases. Also, in this analysis, similar anxiety and depression scores were found, despite comparable levels of pain. Even if we did not find other significant differences in baseline symptom severity and performance status, we cannot exclude the possibility that unmeasured imbalances in quality of life, the extent of disease, or socioeconomic factors might have overshadowed the impact of radiotherapy setting. Maybe, the presence of incurable cancer itself results in a certain burden of anxiety and depression, which is not very dependent on details of therapy. 

Since the patients were not selected for particular primary tumor types or treatment indications, the present study is probably representative of daily clinical practice in palliative radiotherapy facilities such as ours. Nevertheless, the following drawbacks must be taken into account. In retrospective studies, selection bias and incomplete recording of data, including, but not limited to, medications that are prescribed for anxiety or depression, or could cause these symptoms as side effects, might complicate the analyses and their interpretation. With a mean age of more than 70 years, our study group consisted of many elderly and geriatric patients. In addition, the study size was limited. If the present research question was to be addressed in a prospective study, it would have to include about 302 patients (assuming a mean ESAS value of 2.4 and a standard deviation of 3.1 in the first radiotherapy group (see Table 2) and a mean of 1.4 in the reirradiated group; alpha 0.05, beta 0.2, power 0.8). Regarding study limitations, one also has to consider that we did not record the consequences of high symptom scores and their longitudinal changes during and after radiotherapy.

Anxiety was a relevant issue because 38% of reirradiated and 31% of first-time-irradiated patients reported scores ≥4. Regarding depression, 29% of reirradiated and 25% of first-time-irradiated patients reported scores ≥4. The recent ESAS study by Körner et al. reported scores ≥4 for anxiety in 38% and depression in 33% in a palliative radiotherapy setting [6]. A previous study showed that younger patients were significantly more anxious than older patients and females reported more anxiety than males [11]. Patients who reported higher feelings of nausea, tiredness, drowsiness, dyspnea, and worse appetite and overall well-being on the ESAS tool were more likely to report feelings of depression. Patients who reported higher nausea, drowsiness, and dyspnea and worse overall well-being more often reported higher feelings of anxiety. Without a doubt, ESAS is not the most comprehensive and advanced tool for the diagnosis of anxiety and depression [14-16], although it has often been used for the screening of symptoms before radiotherapy [6,11-13,17-18]. It would be interesting to perform additional studies with other tools and more comprehensive questionnaires, and record concomitant medications and pre-existing conditions leading to higher scores.

## Conclusions

The facts that similar ESAS scores of anxiety and depression were observed and that the prognosis was comparable suggest that the magnitude of these symptoms might be associated with the presence of incurable cancer itself (or the related somatic symptom burden) rather than the setting in which palliative radiotherapy is performed.

## References

[1] Chiu N, Chiu L, Popovic M (2015). Re-irradiation for painful bone metastases: evidence-based approach. Ann Palliat Med.

[2] Chow R, Ding K, Ganesh V (2018). Gender and age make no difference in the re-irradiation of painful bone metastases: a secondary analysis of the NCIC CTG SC.20 randomized trial. Radiother Oncol.

[3] Nieder C, De Ruysscher D, Gaspar LE, Guckenberger M, Mehta MP, Cheung P, Sahgal A (2017). Reirradiation of recurrent node-positive non-small cell lung cancer after previous stereotactic radiotherapy for stage I disease: a multi-institutional treatment recommendation. Strahlenther Onkol.

[4] Nieder C, Langendijk JA, Guckenberger M, Grosu AL (2016). Prospective randomized clinical studies involving reirradiation: lessons learned. Strahlenther Onkol.

[5] Mackenzie LJ, Carey M, Sanson-Fisher R, D'Este C, Yoong SL (2015). A cross-sectional study of radiation oncology outpatients' concern about, preferences for, and perceived barriers to discussing anxiety and depression. Psychooncology.

[6] Körner P, Ehrmann K, Hartmannsgruber J, Metz M, Steigerwald S, Flentje M, van Oorschot B (2017). Patient-reported symptoms during radiotherapy: clinically relevant symptom burden in patients treated with palliative and curative intent. Strahlenther Onkol.

[7] Ho RT, Fong TC, Cheung IK, Yip PS, Luk MY (2016). Effects of a short-term dance movement therapy program on symptoms and stress in patients with breast cancer undergoing radiotherapy: a randomized, controlled, single-blind trial. J Pain Symptom Manage.

[8] Halkett GK, O'Connor M, Aranda S (2013). Pilot randomised controlled trial of a radiation therapist-led educational intervention for breast cancer patients prior to commencing radiotherapy. Support Care Cancer.

[9] Chen AM, Vazquez E, Michaud AL, Farwell DG, Purdy JA (2014). Functional and quality-of-life outcomes after reirradiation for head and neck cancer. Laryngoscope.

[10] Nieder C, Astner ST, Mehta MP, Grosu AL, Molls M (2008). Improvement, clinical course, and quality of life after palliative radiotherapy for recurrent glioblastoma. Am J Clin Oncol.

[11] Salvo N, Zeng L, Zhang L (2012). Frequency of reporting and predictive factors for anxiety and depression in patients with advanced cancer. Clin Oncol (R Coll Radiol).

[12] Khan L, Kwong J, Nguyen J (2012). Comparing baseline symptom severity and demographics over two time periods in an outpatient palliative radiotherapy clinic. Support Care Cancer.

[13] Ganesh V, Zhang L, Chan S (2017). An update in symptom clusters using the Edmonton Symptom Assessment System in a palliative radiotherapy clinic. Support Care Cancer.

[14] Rhondali W, Perceau E, Berthiller J (2012). Frequency of depression among oncology outpatients and association with other symptoms. Support Care Cancer.

[15] Nipp RD, El-Jawahri A, Moran SM (2017). The relationship between physical and psychological symptoms and health care utilization in hospitalized patients with advanced cancer. Cancer.

[16] Alfonsson S, Olsson E, Hursti T, Lundh MH, Johansson B (2016). Socio-demographic and clinical variables associated with psychological distress 1 and 3 years after breast cancer diagnosis. Support Care Cancer.

[17] Cheon PM, Pulenzas N, Zhang L (2015). Fatigue scores in patients receiving palliative radiotherapy for painful bone metastases. Support Care Cancer.

[18] Rowbottom L, Chan S, Zhang L (2017). Impact of dyspnea on advanced cancer patients referred to a palliative radiotherapy clinic. Support Care Cancer.

